# What factors influence pain scores following Corticosteroid injection in patients with Greater Trochanteric Pain Syndrome? A systematic review

**DOI:** 10.1186/s12891-024-07217-3

**Published:** 2024-02-17

**Authors:** Ben Foxcroft, Gareth Stephens, Tim Woodhead, Colin Ayre

**Affiliations:** 1 Leeds Institute of Rheumatic and Musculoskeletal Medicine, The University of Leeds, Leeds, UK; 2 Leeds Community Healthcare NHS Trust, Leeds, UK; 3 The University of Bradford, Bradford, UK; 4 The Royal Orthopaedic Hospital NHS Foundation Trust, Birmingham, UK; 5 Bradford Teaching Hospitals NHS Foundation Trust, Duckworth Lane, Bradford, UK

**Keywords:** GTPS, Steroid, Injection, Factors, Outcome

## Abstract

**Background:**

Cortico-Steroid Injections (CSI) are commonly used to treat patients with Greater Trochanteric Pain Syndrome (GTPS) but it is unclear which patients will experience improvements in pain.

**Objectives:**

To identify factors that influence improvements in pain for patients with GTPS treated with CSI.

**Design:**

Systematic review.

**Methods:**

A search was undertaken of AMED, CINAHL, Cochrane Library, EMBASE, Medline and PEDro databases. Studies were eligible for inclusion if they investigated factors that influenced changes in pain experienced by patients with GTPS who received a CSI. Studies needed to include relevant summary statistics and tests of clinical significance. Risk Of Bias in Non-randomised Trials Of Interventions (ROBINS-I) and Risk Of Bias 2 (ROB2) tools were used to assess bias.

**Results:**

The search identified 466 studies, 8 were included in the final review with a total of 643 participants. There was no association between demographic variables such as age, sex, symptom duration or obesity and pain outcomes post-CSI. Having a co-existing musculoskeletal (MSK) condition such as knee osteoarthritis or sacroiliac/lumbar spine pain was associated with less pain reduction post-CSI. Injections into the Trochanteric Bursa were associated with longer lasting pain reduction than Gluteus Medius Bursa or extra-bursal injections. Image guidance of CSI maintained lower pain scores at six months but did not increase the duration of the therapeutic effect past six months. The presence of specific ultrasound scan features was not associated with differences in pain scores.

**Conclusions:**

Patients with co-existing MSK conditions may not respond to CSI as well as those without. Injections into the Greater Trochanteric Bursa may have longer lasting benefit. Further research is needed on the use of USS imaging findings and image guidance.

**Supplementary Information:**

The online version contains supplementary material available at 10.1186/s12891-024-07217-3.

## Background

Greater Trochanteric Pain Syndrome (GTPS) is a debilitating condition presenting as lateral hip pain exacerbated by walking, side-lying, and climbing stairs [27]. It is highly prevalent, affecting up to 25% of women aged over 50 in western populations [63]. The condition is associated with significant pain and functional limitation, leading to high levels of disability and unemployment [20]. The condition was historically referred to as Trochanteric Bursitis, however, the umbrella term of GTPS is now used to reflect the range of pathologies seen on imaging. The imaging features include inflammation of the Trochanteric/Sub-Gluteus Maximus or Sub-Gluteus Medius bursa, tendinopathy of the Gluteus-Medius and/or Gluteus-Minimus and partial or full thickness tears of the Gluteal Tendons. Whilst bursitis continues to be recognised as a pathology, recent evidence shows that tendinopathy of the gluteal tendons is more prevalent than bursitis on imaging [39].

Management of GTPS typically involves education, strengthening exercises and injection therapy [65]. A large randomised controlled trial (RCT) has shown that education and exercise are effective in reducing pain [44] and that Corticosteroid Injections (CSI) provide significant reductions in pain at 12 weeks or less [14]. CSI are commonly used, with an international survey identifying that 40% of physiotherapists recommend their use [25]. When CSI are used, it is often to reduce pain in the short term to provide a window of opportunity to engage in exercise therapy [65]. Guidelines are limited to recommending CSI if symptoms are not improving with conservative management [48]. However, it is clear from the RCT by Mellor et al. [44] that not all patients respond well to CSI with 26/65 (40%) of patients not reporting significant improvements in symptoms at twelve weeks.

Given the variability in patient presentations and injection techniques, an improved understanding of which patients are likely to benefit from CSI would be beneficial for a number of reasons. It would improve shared decision-making and inform patient selection therefore reducing the number of ineffective CSIs. This is important in the context of research into other tendinopathies which has demonstrated adverse events including lower recovery rates and increased recurrences [15] and minimising the risk of infection or anaphylaxis [66]. Finally, considering pressure on time and resources, being able to select the most appropriate patients to receive a CSI would improve the allocation of healthcare resources, a continual area of interest for healthcare providers [37]. Despite the potential benefits of improved outcomes, minimised risk and optimised resource use, there is little guidance for clinicians to aid decision-making regarding which patients with GTPS may respond best to CSI.

## Methods

### Aims

This review aimed to identify factors that influenced changes in pain experienced by patients with GTPS who received a CSI, to provide recommendations for practice. The recommendations will provide clinicians with patient-related factors such as demographics, disease characteristics and imaging findings that may aid in the decision-making around CSI to improve success rates.

### Search strategy

This systematic review followed the Preferred Reporting Items for Systematic Reviews and Meta-Analyses (PRISMA) guidelines [53]. The review was registered on the International Prospective Register of Systematic Reviews (PROSPERO) and the full protocol can be accessed there (CRD42023444138). A systematic search of the following medical and allied-health databases was undertaken: AMED, CINAHL, Cochrane Library, EMBASE, Medline and PEDro. This was complemented by a search of grey literature, Google Scholar, the social media platform Twitter (now known as X), and the reference lists of included articles. Search terms are presented in Table 1. The search was limited to the English language, human studies, and studies that were completed in the last 20 years. All searches took place on 14/04/2023.

**Table 1:** Search strategy

**Search strategy**		
**Databases**	AMED, CINAHL, Cochrane Library, EMBASE, Medline & PEDro	
**Search terms**	**Population** Greater trochanteric pain syndrome OR GTPS OR gluteal tendinopathy OR trochanteric bursitis OR Glut* min* tendinopathy OR Glut* med* tendinopathy OR lateral hip pain OR Hip Burs*	**Exposure** injection* [NEAR/1 or N/1] (corticoid* OR corticosteroid* OR glucocorticoid* OR prednisolone OR methylprednisolone OR triamcinolone acetonide OR triamcinolone hexacetonide OR LACS)
**Additional terms**	English language, Published 2003–2023, human	
**Additional search**	A search of Google Scholar, social media platform Twitter/X, the reference lists of included articles and the available grey literature	

### Study selection

Primary research studies were included if they included patients with GTPS who had received a CSI and measured pain, as a score or a rating of change in pain, as an outcome (see Table 2 for PECOST). Whilst completing Patient and Public Involvement interviews prior to the review, patients and clinicians both reported pain reduction as the main goal of CSI. In a survey of international practice, French et al. [25] found that when physiotherapists use CSI, the main aim was to reduce pain to provide a window of opportunity to exercise. Changes in pain score was therefore used as the primary outcome measure in this review, rather than functional or multi-domain outcome measures. To be eligible for inclusion, studies had to report factors that were associated with the outcomes of CSI, for example, imaging, demographic, or assessment findings, and had to include summary statistics and tests of statistical significance.

**Table 2:** PECOST

**PECOST**	
**Population**	Patients with GTPS
**Exposure**	Corticosteroid injection
**Control**	No control group required
**Outcome measure**	Pain score, Rating of change in pain score
**Study design**	RCTs, Secondary analysis of RCT, cohort studies, observational studies
**Time**	2003—2023

Two reviewers (BF and GS) independently screened titles and abstracts, and potentially relevant abstracts were retrieved for full-text review. There were no conflicts during the title/abstract or full-text review. The inclusion of full-texts was discussed and agreed by the full research team. Where potentially important findings were commented on, in the absence of relevant summary statistics or calculations of statistical significance, a request was made to the authors to provide the relevant information. Both cohort studies and randomised trials can offer valid information when investigating the response to interventions [32], both study designs were therefore included in the review.

### Risk of bias assessment

The Risk Of Bias In Non-randomised Studies of Interventions tool (ROBINS-I) was used to appraise eligible studies. This tool was specifically developed for use with non-randomised studies by the Cochrane Bias Methods Group [67]. For eligible RCTs, the corresponding Risk of Bias 2 (ROB2) tool [68] for randomised trials was used. Two reviewers (BF and GS) independently assessed the risk of bias. There were no discrepancies between assessments. Finally, two reviewers (BF and TW) also completed the Grading of Recommendations, Assessment, Development and Evaluation (GRADE) scoring tool which was used to rate the quality of the body of evidence included in the review [2].

### Data extraction

Data extraction was completed independently by two reviewers (BF and GS) using a modified version of the Cochrane Collaboration data collection form [11]. The data was extracted based on guidelines by the Centre for Reviews & Dissemination [8] and included study and participant characteristics. There were no discrepancies between the reviewers’ extracted findings.

### Analysis

A narrative synthesis was conducted following the Synthesis without Meta-analysis (SWiM) reporting guidelines where possible [7]. A meta-analysis was not conducted due to significant heterogeneity in the methodology, outcome measures, and the variables investigated in each study.

## Results

### Search results

**Fig. 1:**
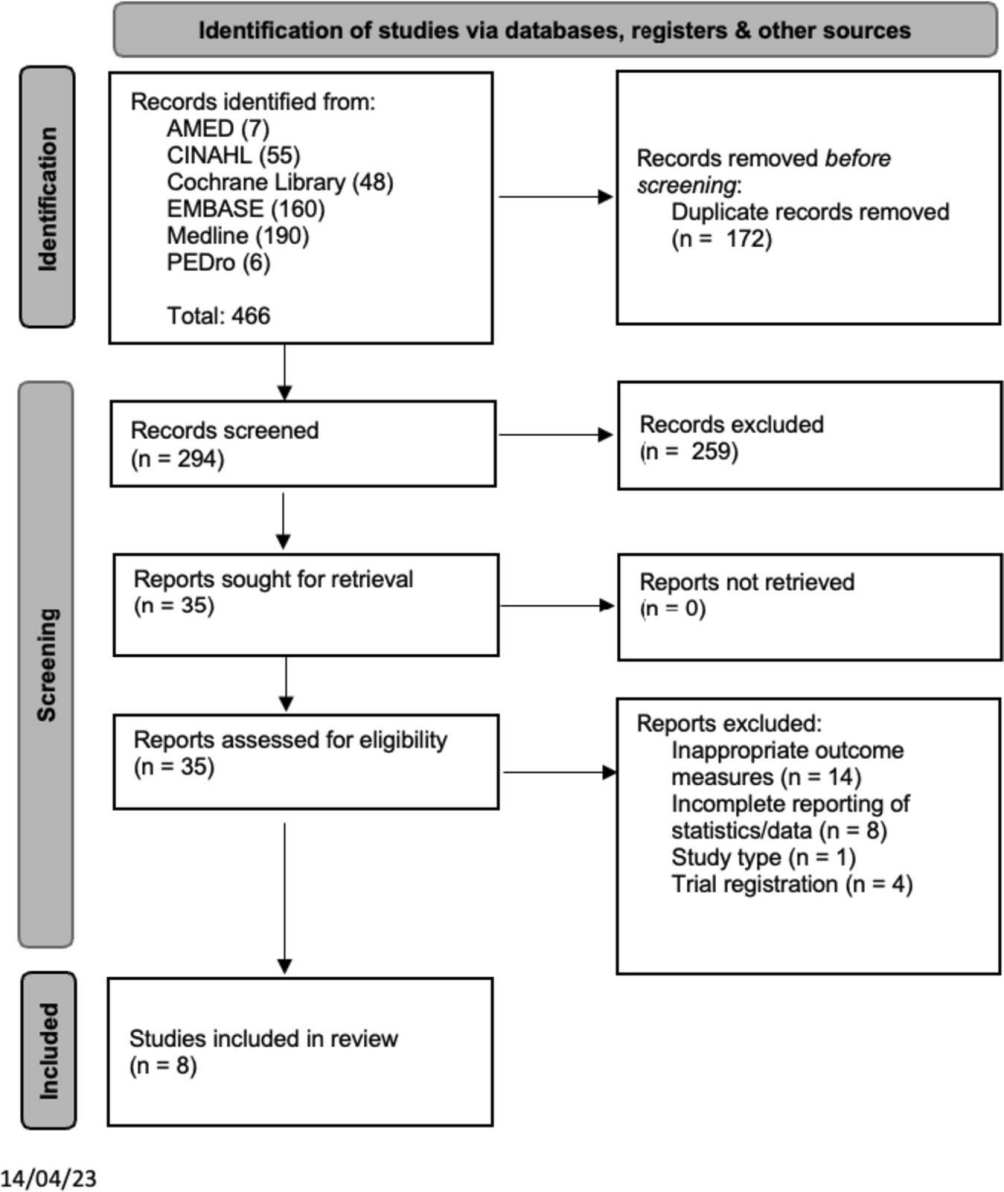
PRISMA flow diagram [53]

The full study selection process is presented in a PRISMA flow diagram (Fig. 1). A total of 294 abstracts were screened for eligibility and 259 were excluded as they did not meet the criteria, leaving 35 requiring full-text review. Of the 35 full-text articles reviewed, 14 were excluded for not investigating factors associated with the outcomes, four for being a trial registration only and one was excluded for being the wrong study type. There were eight articles excluded as they did not contain the appropriate data required to be in the review and it could not be provided despite attempts to contact the authors. The review included eight studies after the selection process. Supplementary file 1 includes the studies excluded after full-text review.

### Quality assessment

**Fig. 2:**
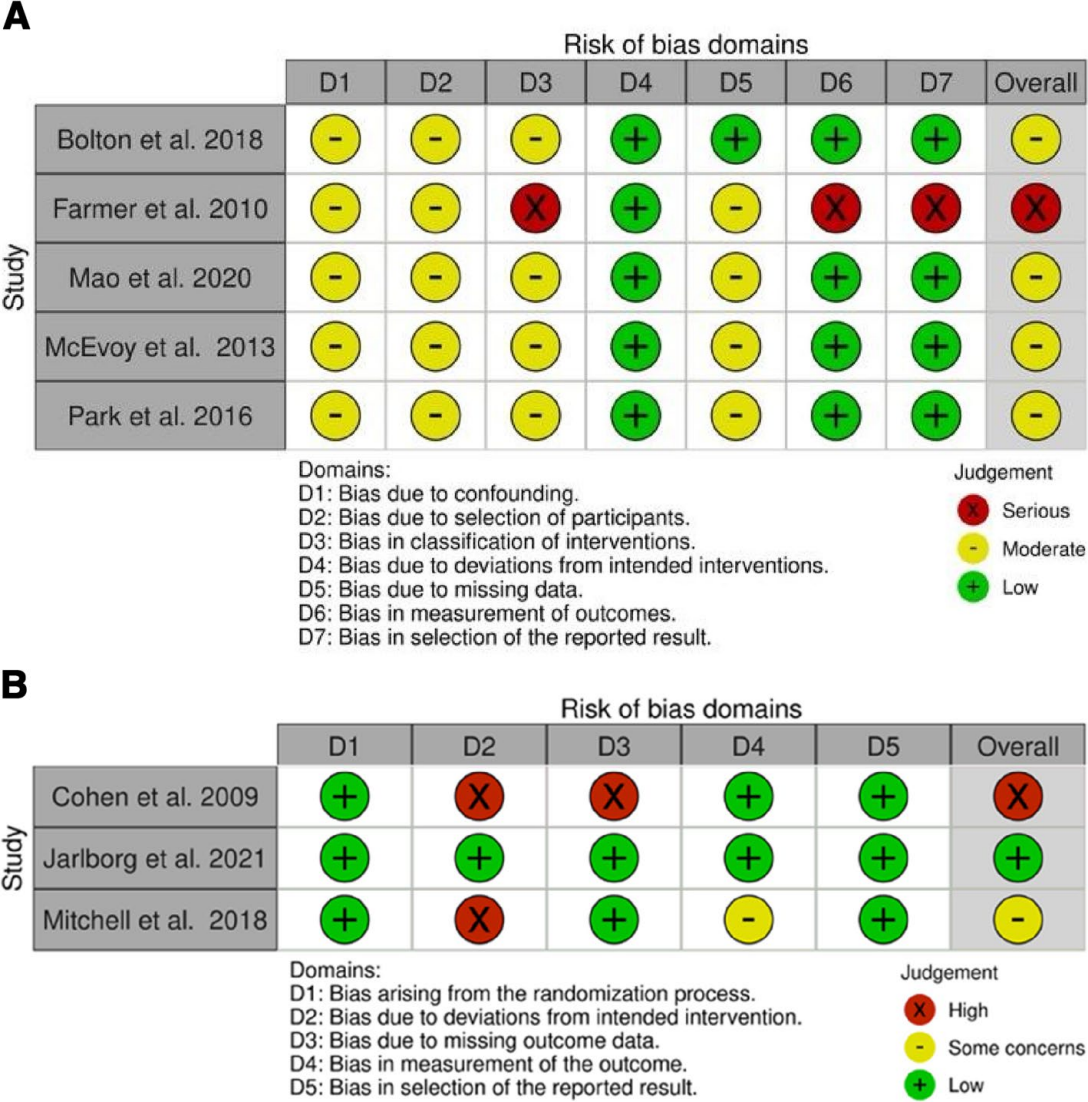
**a** Risk Of Bias In Non-randomised Studies of Interventions (ROBINS-I) traffic light plot. **b** Risk of Bias 2 (ROB2) in randomised trials traffic light plot

The ROBINS-I and ROB2 risk of bias tools were used to assess the methodological quality of the included studies. Of the eight included studies one had a low risk of bias, five had a moderate risk of bias/some concerns and two had a serious/high risk of bias. Risk of bias summaries are presented in Figs. 2a and b. The main areas of concern across the studies were a lack of blinding of investigators, insufficient information on pain measurements and not exploring patient expectations. The review outcomes had low to very low quality of evidence on the GRADE scoring tool – Table 3 presents the scoring summary table.

**Table 3:** GRADE scoring summary table

**Factor investigated**	**Sample**	**Studies**	**Design**	**Limitations**	**Inconsistency**	**Indirectness**	**Imprecision**	**Publication bias**	**Absolute effect (95% CI)**	**Quality**
Patient related factors	227	3	1 RCT 2 R Cohort^a^	Serious limitations^c^	No inconsistency	Serious Indirectness^e^	Serious imprecision^f^	Undetected	Due to heterogeneity of study design, outcome measures and factors investigated meta-analysis was not possible	Low ⨂ ⨂ **◯ ◯**
Image guidance	95	2	2 RCT	Serious limitations^c^	Inconsistency^d^	Serious Indirectness^e^	Serious imprecision^f^	Undetected		Very low ⨂ **◯ ◯ ◯**
Injection location	270	3	1 RCT 2 R Cohort^a^	Serious limitations^c^	Inconsistency^d^	Serious Indirectness^e^	Serious imprecision^f^	Undetected		Very low ⨂ **◯ ◯ ◯**
USS findings	264	2	1 R Cohort^a^ 1 P Cohort^b^	Serious limitations^c^	No inconsistency	Serious Indirectness^e^	Serious imprecision^f^	Undetected		Low ⨂ ⨂ **◯ ◯**

^a^R Cohort = Retrospective cohort

^b^P Cohort = Prospective cohort

^c^Incomplete reporting of data and lack of defined inclusion criteria

^d^Significant unexplained heterogeneity in magnitude of effects

^e^Significant differences in population, intervention, outcome measures and study design

^f^Very wide confidence intervals with different outcomes based on upper and lower limits and small sample

Scoring definitions

High quality: further research is unlikely to change our confidence in the estimate of effect

Moderate quality: further research is likely to have an important impact on our confidence in the estimate of effect

Low quality: further research is very likely to have an important impact on our confidence in the estimate of effect

Very low quality: we are uncertain about the estimate

### Study and participant characteristics

The review included two RCTs, one secondary analysis of an RCT, four retrospective cohort studies and one prospective cohort study. Within the retrospective cohort studies, two investigated factors associated with USS-guided injections, one studied landmark guided and one fluoroscopically guided. The prospective cohort study investigated USS scan findings and guided injections. The two RCTs compared USS-guided and fluoroscopically guided injections to landmark-guided injections. The secondary analysis was of an RCT investigating USS-guided CSI compared to a placebo. The eight included studies investigated 643 participants with a mean age of 57.8 years and 87% were female. Table 4 displays the full study and participant characteristics.

**Table 4:** Study & participant characteristics

**Author, date, country**	**Study design**	**Sample (*N* =)**	**Mean age (years)**	**Sex (% Female)**	**Diagnostic Criteria**	**Intervention**	**Comparator**	**Time frames**	**Primary outcome measure**	**Secondary outcome measures**
Bolton et al. (2018) UK [3]	Prospective cohort	127	63.5	90%	None. Based on the diagnosis of the referring clinician	Ultra-sound guided corticosteroid injections to trochanteric bursa	N/A	Pre-procedure, 6 weeks, 6 months, and 12 months	NRS pain scoring at rest and with activity	Health- Related Quality of Life
Cohen et al. USA [13]	Multi centre double blind randomised controlled study	65	55.2	86.50%	Three months history of insidious onset lateral hip pain. Tender to palpate and one of: pain at maximal hip rotation, abduction or adduction. Pain with forced hip abduction and pseudo-radicular pain down lateral thigh	Fluoroscopically guided trochanteric corticosteroid injections (CSI)	Landmark- guided (blind) cortico-steroid injections (CSI)	Pre-procedure and 1 month post- procedure. 3 months if positive outcome after 1 month	NRS pain at rest and with activity in the last week	SF-36, Oswestry disability index, reduction in drug use, global perceived effect, and a composite successful outcome
Farmer et al. USA [19]	Retrospective cohort	25	Not reported	Not reported	Pain over GT with tenderness on palpation	Landmark-guided corticosteroid injection (CSI)	N/A	No comments on the timing of measurements	Resolution of pain (rating of change)	Harris hip score (HHS), leg length discrepancy, offset of hip, time to injection, number of injections
Jarlborg et al. Switzerland [33]	Secondary analysis of Double blinded placebo controlled randomised trial	44	58.9	83.10%	One month history of lateral hip pain with tenderness on palpation. Pain score over 4 and not improving with conservative management	Ultrasound guided Glucocorticoid injection and local anaesthetic	Placebo injection	Baseline, 30 min, daily for 1 week, weekly for 1 month, 3 months and 6 months	Pain NRS for the previous 24 h period	Oswestry and WOMAC
Mao et al. USA [42]	Retrospective cohort	140	58.2	89.20%	None. Based on the diagnosis of the referring clinician	Fluoroscopically-guided trochanteric corticosteroid injections	N/A	Pre-procedure, immediately post- procedure and 1 week post procedure	VAS score (unspecified^a^)	Nil
McEvoy et al. USA [43]	Retrospective cohort	65	53	86%	Not specific. Clinical diagnosis of GTPS based on the diagnosis of the referring clinician and not improving with conservative management	Ultrasound-guided trochanteric corticosteroid injections	N/A	Pre-procedure, Evening after injection, day 1,2,3,7 and 14	VAS score (unspecified^a^)	Nil
Mitchell et al. USA [46]	Randomised controlled study	40 (30 included in analysis)	50.4	96.50%	Lateral hip pain when walking or at night when side lying, tenderness on palpation, VAS over 5, not improved with conservative management, no tears of Gluteal or other tendons around the hip on USS	Ultrasound-guided corticosteroid injection (CSI)	Landmark-guided CSI	Baseline, during procedure, 2 weeks & 6 months	VAS score (unspecified^a^)	Therapeutic duration, time-to-next intervention, and costs
Park et al. Rep of Korea [54]	Retrospective cohort	137	65.4	78%	Over three month history of lateral hip pain with tenderness on palpation. No improvement with conservative management	Ultrasound-guided trochanteric bursa injection	N/A	Pre-procedure, 1 month, 3 months and 6 months	Verbal Numeric Pain Scale (VNPS) (unspecified^a^) Harris Hip Score (HHS)	Nil

^a^VAS score unspecified: No comment on how the score was asked ie at rest/with activity, at time of asking or other a period of time

### Diagnostic criteria

Bolton et al. [3], Mao et al. [42] and McEvoy et al. [43] did not set a criteria for diagnosing the patient with GTPS. They relied on the clinician making the referral to their radiology department having made an accurate diagnosis. Bolton et al. [3] asked the consultant radiologists providing the injection if they agreed with the referrer diagnosis, which they did 97% of the time. The other studies used a variety of different diagnostic criteria but all of them included lateral hip pain and tenderness on palpation. The full diagnostic criteria for each study can be found in Table 4.

### Outcome measures

All of the studies, apart from Farmer et al. [19], used a Numerical Rating Scale (NRS)/Visual Analogue Scale (VAS) as the primary outcome measure, but there was heterogeneity in its measurement. Bolton et al. [3] recorded pain at rest and with activity. Cohen et al. [13] and Jarlborg et al. [33] both recorded NRS over time but they used different time points with ‘in the last week’ and ‘the last 24 h’ respectively. The remaining studies did not specify how the pain score was determined. Farmer et al. [19] used a rating of change in pain, with the outcome defined as the resolution of pain. The most common time point specified for outcome assessment was six months, this ranged from mid-procedural pain to 12 months across the studies. Table 4 presents all of the outcome measure details.

### Findings

For all findings, *P* values or CI are reported where significant findings were found. Full results are presented in Table 5.

**Table 5:** Results table

Author, date, country	Factors investigated	Factors found	Factors not found
Bolton et al. 2018 UK [3]	USS features of: Greater trochanteric bursitis, Gluteal tendinopathy, Greater trochanteric cortical irregularity, Gluteus Medius bursitis	Nil found	Presence of Greater Trochanteric Cortical Irregularity (GTCI) on USS was, in unadjusted models, associated with a successful outcome in pain with activity at 6 weeks (OR 3.09 [95%CI, 1.06–9.01]; *P* = *0.04*). When the models were adjusted for age, BMI, gender, and evidence of OA the findings were no longer significant (OR 1.21 [95%CI, 0.39–3.73]; *P* = *0.74*) GMB was found also shown to be associated with successful outcomes at 6 weeks at rest, in unadjusted models, (OR 4.49 [95%CI, 1.10–18.36]; *P* = *0.03)* and with activity (OR 4.94 [95%CI, 1.07–22.79]; *P* = *0.03*). Results lost significance when adjusted with (OR 6.10 [95%CI, 0.98–37.91]; *P* = *0.05*) and (OR 6.10 [95%CI, 0.98–37.91]; *P* = *0.05*) with pain at rest and activity respectively No association between GTCI and GMB findings on USS and successful response to injection with reduction in pain score at rest or with activity at 6 months and 12 months in both adjusted and unadjusted models (*P* < *0.06*) No association between GTB or Gluteal tendinopathy and successful response to injection at rest or with activity at 6 weeks, 6 months, and 12 months in both unadjusted and adjusted models (*P* < *0.10*)
Cohen et al. 2009 USA [13]	Blind Vs guided injections, Intra-bursal Vs extra bursal injection location, Patient characteristics such as: age, sex, obesity, duration of pain, opioid use, baseline pain at rest/activity and Oswestry score	Nil found	**Patient characteristics** No differences in mean age (years) between responders 54.6 ± 14.8 and non-responders 55.9 ± 14.4 (*P* = *0.74*) No difference between patient sex with female % at 83% in non-responders and 89% in responders (*P* = *0.72*) No association between obesity and responder status 11/36 (31%) of patients were obese in the non-responder group compared to 6/28 (21%) in the responder group (*P* = *0.57*) No association between baseline pain intensity at rest (mean NRS) in responders 4.7 ± 2.6 compared to non-responders 4.8 ± 2.6 (*P* = *0.26*) No differences in pre-procedure Oswestry disability index scores between responders 42.8 ± 12.6 and non-responders 36.6 ± 14.3 (*P* = *0.46*) No difference between responders and non-responders in pain duration with 5.4 ± 4.5 years and 2.7 ± 2.4 years respectively (*P* = *0.40*) **Injection guidance** No differences in mean pain at one month with rest, fluoroscopically guided 2.7 ± 2.4 and landmark guided 2.2 ± 2.4 (*P* = *0.41*), and with activity 5.0 ± 2.9 compared to 4.0 ± 2.6 (*P* = *0.16*) No differences at three months with fluoroscopically guided mean pain at rest 1.9 ± 1.7 and landmark guided 2.6 ± 2.5 (*P* = *0.34*) and with activity 4.7 ± 2.8 compared to 4.8 ± 2.6 (*P* = *0.90*) There were also no differences in overall success rating (*P* = *0.38*), Oswestry disability index (*P* < *0.68*), global perceived effect (*P* = *0.80*), and analgesia use (*P* = *0.60*) **Injection location** No differences between injection location between intra-bursal or extra-bursal in overall success (*P* = *0.72*), mean pain intensity at rest and with activity at 1 (*P* < *0.53*) and 3 months (*P* < *0.31*). Also no differences in Oswestry score (*P* < *0.45*), global perceived effect (*P* = *0.78*) and analgesia use (*P* = *0.14*)
Farmer et al. 2010 USA [19]	Age, diagnosis pre surgery, approach of hip, leg length discrepancy, offset and lateralisation of replacement and contralateral THR	Nil found	No differences in responder status by age (*P* = *0.1*), diagnosis pre surgery (*P* = *0.4*), approach of hip surgery (*P* = *0.4*), contralateral total hip replacement (*P* = *0.7*), leg length discrepancy (*P* = *0.1*), offset (*P* = *0.3*) and lateralisation (*P* = *0.6*)
Jarlborg et al. 2021 Switzerland [33]	Positive ‘triple test’ = patients with positive tests of Lequesne, Patricks and 30 s single leg stance	Nil found	No significant differences in outcomes between patients with a triple test + ve or -ve (*P* = *0.14*)
Mao et al. 2020 USA [42]	Location of injection: Non- bursal, Subgluteus Medius bursa, and trochanteric bursa	GTB injections maintained the pain reduction between immediate and 1 week with a non-significant change of 0.55 (*P* = *1.0*). Non-bursal and sub-GMB pain scores increased between immediately post injection and 1 week with 1.50 (*P* = *0.012*) and 0.86 (*P* = *0.014*) respectively	No differences between injection location between sub-GMB (*P* = *0.46*), GTB (*P* = *1.0*) and non-bursal (*P* = *1.0*) immediately post procedure or at 1 week (*P* = *1.0*)
McEvoy et al. 2013 USA [43]	Injection location of GMB Vs GTB	GTB injections resulted in statistically significant reduction in median pain scores of 3.0 (*P* < *0.01*) compared to sub-GMB injections with non-significant reductions of 0 (*P* = *0.44*) and a significant difference between the two injection locations. (*P* < *0.01*)	Nil
Mitchell et al. 2018 USA [46]	Ultrasound guided Vs landmark guided	Significant differences between groups in pain score at 6 months; mean pain scores with USS 3.9 ± 2.0 landmark guided 5.5 ± 2.6 (*P* = *0.036*)	No significant difference in mean pain scores at 2 weeks post CSI: USS 1.3 ± 1.9 and landmark 2.2 ± 2.5 (*P* = *0.61*) No differences in procedural pain; USS 3.3 ± 2.3 and landmark 4.9 ± 4.1 (*P* = *0.20*) No differences in time to next intervention (months) USS 8.7 ± 2.9 and landmark 8.3 ± 3.8 (*P* = *0.75*) No differences in duration of therapeutic effect (months): USS 4.7 ± 1.4 and landmark 4.1 ± 2.9 (*P* = *0.48*)
Park et al. 2016 Rep of Korea [54]	Patient characteristics: Age, sex, pain duration, co-morbidity, BMI, presence of bursitis, tendinosis, enthesitis or partial/full thickness tears on USS	Worse outcomes associated with having a co-existing MSK condition of knee OA (OR 0.329 [95%CI, 0.128–0.848]; *P* = *0.01*) and Sacro-Illiac Joint (SIJ) or lumbar spine pain (OR 0.304 [95%CI, 0.118 – 0.783]; *P* = *0.01*)	No association between pain reduction and age (*P* = *0.661*), gender (*P* = *0.558*), BMI (*P* = *0.172*) and pain duration (*P* = *0.187*) No association between pain reduction and imaging findings of tendinosis OR 3.17 [95%CI, 0.89–11.33], bursitis OR 4.20 [95%CI, 0.87–20.33], partial tear OR 2.94 [95%CI, 0.76–10.90], full thickness tear OR 3.27 [95%CI, 0.85–12.63] and enthesopathy OR 3.82 [95%CI, 0.81–18.03]

### Patient characteristics

Three studies [13, 19, 54] examined the patients’ age and the response to CSI,none of the studies found any statistically significant association. Two studies [13, 54] investigated the influence of the patients’ sex and the duration of symptoms on the response to injections and neither of the studies found any differences in response based on these factors. They also investigated the impact of obesity on injection outcomes. Obesity was defined in both trials as a Body Mass Index (BMI) of over 30 kg/m2. Neither trial found any significant differences between responders and non-responders based on BMI. Cohen et al. [13] also found no association between baseline pain intensity and rating of pain post-injection. Park et al. [54] investigated the association between having co-existing musculoskeletal (MSK) conditions and being a ‘responder’, defined as having a reduction in their pain score of over 50%. Having a diagnosis of knee osteoarthritis (OA) was associated with being a non-responder (OR 0.329 [95%CI, 0.128–0.848]; *P* = 0.01), whilst having co-existing Sacroiliac Joint (SIJ) or lumbar spine pain was associated with being a non-responder (OR 0.304 [95%CI, 0.118 – 0.783]; *P* = 0.01).

### Image guidance

Image guidance of CSI using fluoroscopy and USS was investigated by Cohen et al. [13] and Mitchell et al. [46] respectively. Fluoroscopic guidance was found to offer no significant benefits in pain scores at one and three months compared to landmark guidance but increased costs by over 547% ($1216 vs $188 respectively). USS guidance provided no significant difference in pain reduction from baseline at two weeks compared to landmark guidance but did maintain the benefit better at six months. This maintained change was statistically and clinically significant, with landmark guidance VAS scores at 5.5 ± 2.6 compared to USS guidance at 3.9 ± 2.0 with a difference of 34% (*P* = 0.036). There was no benefit in the duration of the therapeutic effect past six months and it did not change the time-to-next intervention. The use of USS significantly increases costs with a 30% increase, USS $297 vs landmark $207 (*P* = 0.017).

### Ultrasound scan findings

Two studies investigated the presence of imaging findings on USS and whether this affected the outcome of the injection. The imaging features investigated by the studies were the presence of bursitis in the Greater Trochanteric Bursa (GTB) or Gluteus Medius Bursa (GMB), tendinopathy/tendinosis, enthesitis/enthesopathy/cortical irregularity, and Park et al. [54] also included partial/full thickness tears. Park et al. [54] found no association between imaging findings and outcomes. Bolton et al. [3] found no association between GTB and gluteal tendinopathy on outcomes at rest or with activity at six weeks, six months, and 12 months in both unadjusted and adjusted models. They did find that the presence of GMB and Greater Trochanteric Cortical Irregularity (GTCI) was associated with successful outcomes at six weeks, in unadjusted models. These findings were not significant when adjusted for age, BMI, gender and having a diagnosis of hip or knee OA. Neither GTCI or GMB were associated with changes in outcomes at any other time point.

### Location of injection

Three studies explored the effect of steroid injection location on outcomes, they classified this into the GTB, sub-GMB or non-bursal. Cohen et al. [13] found no differences in overall success, mean pain intensity at rest and with activity at one and three months. Mao et al. [42] found no differences between sub-GMB, GTB and non-bursal immediately post-procedure or one week after. They did find that while non-bursal and sub-GMB pain scores began to rise over the week post-injection, GTB injection pain scores maintained the pain reduction better with no significant change after a week. McEvoy et al. [43] also found that injections into the GTB resulted in a significantly larger reduction in pain compared to sub-GMB (*P* < 0.01). GTB injections had a reduction in median pain score of 3.0 (*P* < 0.01) compared to sub-GMB with a median pain reduction of 0 (*P* = 0.44).

## Discussion

This review aimed to establish factors associated with the effectiveness of CSI, based on pain scores, in patients with GTPS. There was a paucity of high-quality evidence identified, precluding definitive recommendations for clinical practice. However, it was possible to identify factors that have been shown to influence the effectiveness of CSI in reducing pain and others which do not.

None of the studies found any links between successful injection outcomes and age, sex, obesity, duration of symptoms or physical exam findings. These factors should not, therefore, influence the selection of patients for CSI based on current evidence. Cohen et al. [13] did not find any association between patient demographics and response to CSI. However, the removal of non-responders from the study at 1 month may have reduced the likelihood of them finding a negative association in their analysis of responder status at three months. Patient related factors have been investigated as predictors of response to CSI in other patient groups. In a secondary analysis of an RCT, Whittaker et al. [71] found similar results to this review with no difference in outcomes based on age, BMI, and sex for patients with plantar heel pain. They did find that patients who did not weight-bear as much and who had lower baseline pain scores had improved outcomes post CSI. Jerosch-Herold et al. [34] found that response to CSI in patients with Carpal Tunnel Syndrome was improved if they had a shorter duration of symptoms and had no prior CSI. In keeping with our findings, they also found no differences in response with respect to age, BMI and sex.

There were significant findings regarding co-existing MSK conditions. Park et al. [54] found that patients with knee OA or lumbar spine/SIJ pain experienced less pain reduction in their GTPS symptoms after CSI compared to those without. This information may be useful to provide patients with accurate information regarding the likelihood of success of CSI when engaging in shared decision-making, particularly if the patient is uncertain about the procedure. There may be many reasons for patients with co-existing MSK conditions not responding as well to CSI. Firstly, there is still uncertainty about the pain mechanisms that underpin GTPS and the mechanism of action of CSI. Theories about its mechanism of action are based around the inhibition of pro-inflammatory cytokines or neuropeptides, such as Calcitonin Gene Related Peptide (CGRP), a mediator of neurogenic inflammation [29]. These mechanisms are local tissue orientated and it is unclear how CSI would benefit patients who present with evidence of central sensitisation, an amplification of signalling within the central nervous system [49]. Patients with central sensitisation often have multiple sites of pain [49] and have worse outcomes after corticosteroid injections [26], physiotherapy [51] and surgery [36]. French et al. [24] found up to 44% of patients with GTPS had evidence of central sensitisation. Second, patients often have altered biomechanics when they suffer with spinal pain [1] or knee osteoarthritis [47]. Sustained alterations in biomechanics and subsequent impact on kinematics throughout the lower extremity [62] may negate the effects of CSI. Finally, the lumbar spine and pelvic region has multiple sources of pain which can have overlapping referral patterns [40] making an accurate diagnosis more complex [57]. In patients with multiple sites of pain, local CSI injection may be expected to be less effective in alleviating pain.

Mitchell et al. [46] found that when compared to landmark guidance, USS-guided injections did not improve pain scores at two weeks and did not increase the total duration of therapeutic effect or the time to the patient next seeking intervention. They did find that injections under USS guidance were associated with maintained pain reduction at six months. This pain reduction was above the 'minimal important difference' for MSK conditions, but below the cut-off point for ‘much better’ proposed by Salaffi et al. [60]. When interpreting the findings of Mitchell et al. [46] several considerations should be made. Firstly, the study may have been underpowered to identify the benefits of USS guidance with a trend toward improvements in pain both during the procedure and at two weeks following. The authors conducted a power calculation and identified that they would need to include 150 participants to demonstrate a statistically significant difference between groups – they included 30 participants in the trial. However, the failure to blind patients, and an inability to blind clinicians, may have biased results in favour of the USS group with previous evidence showing the majority of patients believe this is superior to landmark-guided injections [17].

USS-guided injections may offer improvements in pain reduction over a longer period of time, but this is based on limited evidence. It is unclear if the benefits found in this study justify the increase in costs from an economic perspective with USS guidance increasing costs by 30% compared to landmark guidance. Based on both of these issues it is not possible to routinely recommend the use of image guidance when injecting CSI in patients with GTPS, without further research. A Cochrane review by Zadro et al. [73] sought to answer a similar question regarding the use of image-guided CSI compared to landmark-guided for shoulder pain. They found with moderate certainty evidence that the use of USS provided little or no benefit over landmark guidance on pain and quality of life. They also found it did not reduce the risk of adverse events. They concluded that the lack of significant benefit suggests that the extra associated costs were not justified.

Two of the three studies investigating injection location found that injections into the GTB were either more effective or longer lasting than sub-GMB or non-bursal injections. Mao et al. [42] and McEvoy et al. [43] showed early benefit over the other injection locations, however, both studies failed to report outcomes in the medium to longer term. As it is currently unclear if this benefit was sustained beyond the first week or two post injection, the findings are of limited clinical relevance. However, based on this limited evidence, clinicians should aim to inject into the GTB. Further research with longer-term follow-up is required. Cohen et al. [13] found no association between injection location and outcomes. This was an RCT but had a much smaller sample size and using fluoroscopy, only compared intra-bursal and extra-bursal injections and not the specific bursa, which may explain their contradictory findings. In other patient groups, a secondary analysis of an RCT investigating USS-guided injections into the shoulder for Sub-acromial pain syndrome found that there was no difference in pain or function dependent on the location of injection [9]. A further RCT investigating injection location for Osteoarthritis of the knee joint also found no differences in function or pain between different injection location sites [16].

The presence of ultrasound findings and their association with outcomes was investigated by two studies, with conflicting results. Park et al. [54] found no statistically significant findings. In contrast, Bolton et al. [3] found that Gluteus Medius Bursitis, in unadjusted models, was associated with improved outcomes in pain with activity at six weeks OR 4.94 [95%CI, 1.07-22.79] *P* = 0.03. These findings should be interpreted with caution though as when adjusting for age, sex, BMI and co-morbidity they lost significance. Furthermore, the wide confidence intervals suggest the study was underpowered and therefore its findings lack precision. However, the point estimate and upper limit of the confidence interval suggests there could be a possibility of a meaningful association and with a potentially underpowered study this may warrant further research. Investigations of CSI for other conditions have found significant imaging features which can be used as predictors of outcomes. Breton et al. [5] found that patients with plantar heel pain whose plantar fascia was thicker than 7 mm on USS had better outcomes from CSI at 6 months. Another study investigating Carpal Tunnel Syndrome found that patients whose median nerve had a thicker cross-sectional area on USS were also more likely to respond to CSI [10].

This systematic search of the available literature and appraisal of this found a relatively small number of heterogenous trials that were mostly of moderate quality and had some significant methodological issues such as a lack of blinding of patients and examiners. Lack of blinding of the patients may introduce performance bias, which may favour the intervention based on patient expectations/beliefs. None of the studies investigated patient beliefs/expectations, which would have allowed for appraisal of performance bias. Lack of blinding of outcome assessors may introduce detection bias and this may influence the results based on factors that were not controlled for in the studies. These issues do, however, reflect the nature of administering CSI in clinical practice and can therefore be considered a pragmatic investigation of the intervention [55]. Another common area of concern of the studies included in the review was the use of multiple comparison tests. Conducting multiple comparisons simultaneously can lead to false positive findings, as for each additional test the likelihood of attributing significance to random variability increases [21]. Mao et al. [42] was the only study to use a Bonferroni correction which reduces the likelihood that their findings were false positives and a result of type 1 errors [52].

### Strengths and limitations of this review

This review is the first to systematically search the literature and appraise the evidence base to identify factors that may influence the response to steroid injection for patients with GTPS. It is limited by the lack of high-quality evidence. There were some statistically significant findings identified during the literature review which may guide further research but at present it is not possible to make definitive recommendations. Further, a meta-analysis was not possible in this review due to heterogeneity in outcome measures. The review was strengthened by having two independent reviewers completing all of the study selection, data extraction, risk of bias and GRADE scoring.

### Implications for practice and further research

When considering the use of CSI clinicians should be mindful of the risk-to-reward profile in patients with co-existing MSK conditions who may not respond as well to CSI and patients should be counselled on this. This systematic review suggests that when used, injections should be completed with landmark guidance and aim to inject the GTB. Whilst there is evidence injections may be more effective into the GTB, at least in the short term, there is uncertainty as to the long-term significance of this. The use of USS to guide injections provided a clinically significant benefit at 6 months but based on the limited quality of evidence and the increased costs of USS it is not recommended that this is used routinely over landmark guidance. Further RCTs with appropriate sample sizes and built in economic evaluations would help to establish its benefit and if the associated increase in costs are justified. The use of USS findings to guide decision-making requires further research. To build upon the recommendations of this review, future research studies should utilise a prospective cohort methodology with a large enough sample size to address the question. A pragmatic approach should be taken and specific areas to improve are assessor blinding and collecting data on all the potential factors involved within one trial. At the time of writing, there is no defined core outcome set for GTPS. Establishing this would ensure studies use appropriate standardised outcome measures and allow for meta-analysis in future reviews.

## Conclusions

This is the first systematic review to investigate factors associated with the response to CSI. There is a lack of high-quality studies, but based on the limited evidence, there are three key findings. Firstly, there was no evidence of an association between outcomes and patient characteristics such as age, sex, duration of symptoms and obesity, but patients with co-existing MSK conditions such as knee OA or spinal pain may not respond as well. Secondly, there is evidence injecting CSI into the GTB may be associated with larger and longer-lasting pain reductions. Within the limits of current evidence, it is reasonable for clinicians to consider GTB injection over sub-GMB or non-bursal injections. Finally, further research is needed to investigate the use of imaging features to aid in decision making and the use of USS guidance of injections.

### Supplementary Information

**Additional file 1: Supplementary file 1.** Studies excluded after full text review [4, 6, 12, 18, 22, 23, 28, 30, 31, 35, 38, 41, 45, 50, 56, 58, 59, 61, 64, 69, 70, 72].

## Data Availability

The datasets used and/or analysed during the current study are available from the corresponding author on reasonable request.

## Abbreviations

BMI: Body Mass Index
CI: Confidence Intervals
CSI: Corticosteroid injection
GMB: Gluteus Medius Bursa
GTB: Greater Trochanteric Bursa
GTCI: Greater Trochanteric Cortical Irregularity
GTPS: Greater Trochanteric Pain Syndrome
LACS: Local Anaesthetic Corticosteroid
MSK: Musculoskeletal
NRS: Numerical Rating Scale
OA: Osteoarthritis
OR: Odds Ratio
PRISMA: Preferred Reporting Items for Systematic Reviews and Meta-Analyses
RCT: Randomised Controlled Trial
ROB2: Risk of Bias 2
ROBINS: Risk of Bias In Non-randomised Studies
SIJ: Sacro-illiac Joint
USS: Ultrasound Scan
VAS: Visual Analogue Score
VNPS: Visual-Numeric Pain Score
WOMAC: Western Ontario and McMaster Universities Osteoarthritis Index
