# Supplementary material for: What factors influence pain scores following Corticosteroid injection in patients with Greater Trochanteric Pain Syndrome? A systematic review
Source: BMC Musculoskelet Disord. 2024 Feb 17;25:149. doi: 10.1186/s12891-024-07217-3 (PMC10874005; doi:10.1186/s12891-024-07217-3)
Supplement: Supplementary file 1 — Additional file 1: Supplementary file 1. Studies excluded after full text review [4, 6, 12, 18, 22, 23, 28, 30, 31, 35, 38, 41, 45, 50, 56, 58, 59, 61, 64, 69, 70, 72]. [file 12891_2024_7217_MOESM1_ESM.docx]

Supplementary file one: Studies excluded after full text review

| **Reference** | **Reason for exclusion** |
| --- | --- |
| Brennan et al. 2017 | Incomplete reporting of statistics/data |
| Brinks et al. 2011 | Incomplete reporting of statistics/data |
| Cohen et al. 2005 | Inappropriate outcome measures: did not investigate *factors* associated with outcomes. |
| Estrela et al. 2014 | Inappropriate outcome measures: did not investigate *factors* associated with outcomes. |
| Ferrari 2012 | Inappropriate outcome measures: did not investigate *factors* associated with outcomes. |
| Fitzpatrick et al. 2018 | Incomplete reporting of statistics/data |
| Guillen-Astete et al. 2014 | Inappropriate outcome measures: did not investigate *factors* associated with outcomes. |
| Heaver et al. 2021 | Inappropriate outcome measures: did not investigate *factors* associated with outcomes. |
| Imran et al. 2014 | Inappropriate outcome measures: did not investigate *factors* associated with outcomes. |
| Kargela et al. 2017 | Incorrect study type: *Not primary research* |
| Labrosse et al. 2010 | Incomplete reporting of statistics/data |
| Lievense et al. 2005 | Inappropriate outcome measures: did not investigate *factors* associated with outcomes. |
| Mellor et al. 2018 | Inappropriate outcome measures: did not investigate *factors* associated with outcomes. |
| Mellor et al. 2022 | Inappropriate outcome measures: did not investigate *within CSI group* factors |
| Nissen et al. 2019 | Incomplete reporting of statistics/data |
| Pereira et al. 2015 | Inappropriate outcome measures: did not investigate *factors* associated with outcomes. |
| Rompe et al. 2009 | Inappropriate outcome measures: did not investigate *factors* associated with outcomes. |
| Rosário et al. 2021 | Inappropriate outcome measures: did not investigate *factors* associated with outcomes. |
| Schlesinger et al. 2013 | Inappropriate outcome measures: did not investigate *factors* associated with outcomes. |
| Skibicki et al. 2021 | Incomplete reporting of statistics/data |
| Walker et al. 2007 | Incomplete reporting of statistics/data |
| Wilson et al. 2014 | Incomplete reporting of statistics/data |
| Whetsell et al. 2012 | Inappropriate outcome measures: did not investigate *factors* associated with outcomes. |
